# P076: Analysis of resistance, virulence, and phylogenetic groups of Escherichia coli strains isolated from very-low birth weight infants in Poland

**DOI:** 10.1186/2047-2994-2-S1-P76

**Published:** 2013-06-20

**Authors:** A Chmielarczyk, M Pobiega, D Romaniszyn, J Wojkowska-Mach, P Heczko

**Affiliations:** 1 Microbiology, Jagiellonian University Medical School, Kraków, Poland

## Objectives

Infections of the newborns remain one of the most significant problems of the medicine. This project aims to determine relationship between illness, resistance, virulence factors (VFs), phylogenetic groups and genotypes among E.coli isolates coming from 6 Polish Neonatal Intensive Care Units, NICUs.

## Methods

A total of 90 E.coli (EC) were collected in 2009-2011. Isolates coming from bloodstream infections (BSI) 24, respiratory tract (RT) 50, urinary tract infections -8 and others 8 were tested.

Species was determined with the Vitek system, drug resistance was determined by disc diffusion method (according to EUCAST). The nucleotide sequences of beta-lactamases were obtained. Isolates were checked for the presence of 16 selected VF genes associated with extraintestinal infections and classified into 4 ECOR groups using PCR. Clone ST131 was detected. Genotyping was carried out using PFGE.

## Results

The EC infection incidence was 9.3%. ESBL activity was detected in 25 isolates. Among them 16 were resistant to at least two other groups of antibiotics. All ESBL isolates carried the bla-CTX-M gene (60% had CTX-M-15 and 40% had CTX-M-3) and 12 isolates had also TEM-1 gene. The clone ST131 was detected in 33 strains and 12 of them carried the CTX-M-15 gene.

Most frequently detected adhesion genes were fimH 75% papC 51% and sfa 44%. From iron-related genes often occurred: fhuA 87%, fecA75%, fyuA 56%, iroN 55%, iutA 54%.

The blood isolates were more likely to possess the ibeA than were isolates from urine and RT (37,5%, 0%, 16% respectively), and urine isolates - iha (75% vs. 24% - RT vs. 8.3% - blood).

7 isolates clustered in ECOR group A, 5 in B1, 62 - in B2 and 16 in D group. In B2 group the number of VFs was higher than in A and D (29% isolates from B2 group had >11 VFs and 56% isolates from A and D group had <3 VFs).

The majority of the isolates belonging to the ST131 clone were from the B2 group,

EC isolates showed very different pulsotypes, epidemic clones were not detected.

## Conclusion

EC strains contribute significantly to late RT infections and BSI in NICUs. More than 1/4 of EC strains showed ESBL phenotype. Most of the strains belonged to the group B2 (68.8%) and had numerous VFs. EC isolates showed high genetic diversity.

2011/01/D/NZ7/00104

## Disclosure of interest

None declared

